# Adipose Reserves and Survival: Protective in Prefrail but Ineffective in Frail Oldest‐Old

**DOI:** 10.1002/jcsm.70358

**Published:** 2026-08-13

**Authors:** Jin Yan, Ming Zhao, Cheng Qin, Zhihui Zhu, Ning Di, Bingjie Lu, Cunmei Yang, Qiuli Xu, Zheng Liu, Li Fan, Yixin Hu

**Affiliations:** ^1^ The Second Medical Center & National Clinical Research Center for Geriatric Diseases Chinese PLA General Hospital Beijing China; ^2^ The Fourth Comprehensive Service Guarantee Center of the Veteran Cadre Service Administration of the Beijing Garrison District Beijing China; ^3^ Quzhou Experimental School Education Group Quzhou China; ^4^ Baoding University of Technology Baoding China; ^5^ Mudanjiang Hospital of Beidahuang Group Mishan China

**Keywords:** adipose reserves, all‐cause mortality, frailty, obesity paradox, oldest‐old

## Abstract

**Background and Aim:**

Among the oldest‐old, the impact of body composition on survival outcomes exhibits pronounced heterogeneity. Although the obesity paradox has been widely discussed, clear evidence is still lacking regarding whether the biological effects of adipose reserves are modulated by functional status. Therefore, this study aims to investigate the association between adipose reserve indicators and long‐term all‐cause mortality risk in the oldest‐old and to elucidate the regulatory role of frailty, thereby providing an evidence‐based foundation for precision clinical health management.

**Methods:**

This prospective cohort study included 529 community‐dwelling oldest‐old individuals. Participants were categorized into prefrailty and frailty groups at baseline using the Fried frailty phenotype. Bioelectrical impedance analysis was utilized to measure body composition. Stratified multivariate Cox proportional hazards regression models were employed to evaluate the relationship between adipose indicators and all‐cause mortality. Additionally, restricted cubic splines (RCS) and Kaplan–Meier curves were used to analyse survival heterogeneity.

**Results:**

During a median follow‐up of 3.84 years, 88 deaths (16.6%) occurred. In the prefrail oldest‐old, adipose reserves exhibited significant protective effects on survival: The fully adjusted model showed that PBF (HR = 0.92, 95% CI: 0.87–0.98, *p* = 0.006), VFA (HR = 0.99, 95% CI: 0.97–0.99, *p* = 0.013) and WHR (HR = 0.91, 95% CI: 0.86–0.95, *p* < 0.001) were all inversely associated with mortality risk. In contrast, these protective effects vanished in the frail oldest‐old (all *p* > 0.05), suggesting that the benefits of adipose tissue are contingent upon the individual's basal functional reserve. RCS analysis revealed a linear dose–response relationship between adipose indicators and mortality risk (all *p* for non‐linearity > 0.05), with no significant threshold effect observed. Interaction analyses confirmed that frailty status significantly modulated the prognostic value of adipose metrics (*p* < 0.05), with protective effects restricted exclusively to prefrail individuals. Kaplan–Meier curves confirmed that individuals in the prefrailty stage with higher adipose reserves achieved the highest survival rates (all Log‐rank *p* < 0.001).

**Conclusion:**

These findings suggest that the protective effect of adipose reserves is frailty‐dependent in the oldest‐old, and that weight management strategies should consider baseline functional status.

## 1 Introduction

With the intensifying global ageing of the population, the oldest‐old (individuals aged 80 and above) have emerged as a focal point in healthcare research [1]. This population exhibits pronounced heterogeneity in terms of physiological reserve, pathological characteristics and metabolic patterns; consequently, conventional clinical risk assessment models often struggle to provide accurate long‐term prognostic predictions. During the process of ageing in extreme longevity, the decline of multisystem functions is typically accompanied by a remodelling of body composition [2]. How to precisely define the relationship between body composition indicators and survival risk remains a formidable challenge in the field of geriatrics.

For a long time, the loss of skeletal muscle mass has been widely regarded by the academic community as a critical marker of functional impairment and increased mortality risk in the oldest‐old [3]. However, the role of adipose tissue in this process remains elusive [4]. On the one hand, central obesity is traditionally considered a major risk factor for metabolic disorders and cardiovascular events [5, 6]. On the other hand, research on the obesity paradox in the elderly suggests that higher levels of body fat may be associated with survival benefits [7]; specifically, a substantial 4‐year decline in obesity indicators was associated with elevated mortality risks in older adults [8]. This phenomenon implies that, in the stage of extreme longevity, adipose tissue may transition from a traditional metabolic burden to a form of vital energy reserve. Nevertheless, it remains unclear whether this transition exhibits specific heterogeneity across different disease states.

In addition, frailty serves as a pivotal metric of integrated physiological integrity, capturing the organismal vulnerability to external stressors. While previous research has examined the link between body composition and clinical outcomes [9], it remains systematically unproven whether the biological implications of fat stores are contingent upon the individuals' functional baseline—most notably, the severity of frailty. This suggests that the purported protective role of adiposity may be heterogeneously expressed across different frailty phenotypes.

Consequently, utilizing a 4‐year prospective cohort design, the present study seeks to delineate the nexus between adipose stores, skeletal muscle metrics and long‐term all‐cause mortality in the community‐dwelling oldest‐old. With a specific focus on whether frailty status exerts a moderating effect on this association. This study attempts to clarify whether the survival‐protective effect of adipose reserves exhibits population heterogeneity and whether such protection involves a specific functional compensatory threshold. By addressing these questions, we expect to provide more targeted, evidence‐based guidance for individualized weight management in the oldest‐old population.

## 2 Methods

### 2.1 Study Design and Participants

This prospective cohort study was conducted between January and June 2019 at a community health service centre in Haidian District, Beijing. Eligibility criteria required participants to be (1) aged 80 years or older; (2) adjudicated as ‘pre‐frail’ or ‘frail’ according to the Fried frailty phenotype; and (3) willing and able to comply with all study assessments. Individuals were precluded from the study if they (1) had contraindications for or refusal of body composition measurements; (2) exhibited severe communication barriers due to advanced cognitive decline, dementia, profound hearing loss or psychiatric conditions; or (3) were in the terminal stages of illness or a wasting state with an anticipated survival of less than 1 year.

The primary endpoint was all‐cause mortality, with the longitudinal follow‐up conducted from January to April 2023. Mortality events and exact dates of death were identified and verified through a combination of telephone interviews with participants or their next of kin and an extensive review of electronic medical records.

The study protocol received ethical clearance from the Ethics Committee of the Chinese People's Liberation Army General Hospital (Approval No. S2018‐102‐02) and was prospectively registered with the Chinese Clinical Trial Registry (Registration No.: ChiCTR900022576). All data were de‐identified to ensure anonymity, and written informed consent was obtained from all participants prior to enrolment.

### 2.2 Assessment

#### 2.2.1 Frailty Phenotype

Frailty status was evaluated using the Fried frailty phenotype, established by Fried et al. [10] from the Johns Hopkins University School of Medicine in 2001. This instrument assesses five core components: (1) unintentional weight loss, (2) exhaustion, (3) low physical activity, (4) slowness and (5) weakness. Each criterion is scored as 1 for ‘Yes’ and 0 for ‘No’. The cumulative score ranges from 0 to 5, based on which participants are categorized as robust (score of 0), prefrail (score of 1–2) or frail (score of 3–5).

#### 2.2.2 Body Composition

Body composition was assessed via bioelectrical impedance analysis (BIA) using the InBody 770 analyser (Biospace, Seoul, South Korea). Prior to measurement, participants were screened for metallic implants or cardiac pacemakers. Following standard protocols, participants were instructed to remove shoes and socks, stand barefoot on the foot electrodes with their heels aligned with the base and grasp the hand electrodes. Proper positioning required the thumbs to rest on the upper electrodes, whereas the other fingers gripped the lower electrodes, with arms extended and slightly abducted from the torso throughout the automated test. Key metrics included body weight, body mass index (BMI), percentage of body fat (PBF), visceral fat area (VFA), waist‐to‐hip ratio (WHR), skeletal muscle index (SMI) and segmental muscle mass (limbs and trunk). Additionally, participants were stratified according to Chinese‐specific BMI cut‐offs [11]: underweight (< 18.5 kg/m^2^), normal weight (18.5–23.9 kg/m^2^), overweight (24.0–27.9 kg/m^2^) and obese (≥ 28.0 kg/m^2^).

#### 2.2.3 Covariates

Comprehensive data were collected through standardized one‐on‐one structured interviews and clinical assessments. Sociodemographic characteristics included age, sex, ethnicity, educational attainment, marital status (married, divorced or widowed), monthly income and caregiving status. Lifestyle behaviours were assessed based on smoking and alcohol consumption habits within the past 12 months. Medication use was documented, with polypharmacy defined as the concurrent use of five or more medications [12]. Comorbidities were recorded as the presence of physician‐diagnosed chronic conditions.

Comorbidities were ascertained based on a combination of physician‐diagnosed chronic conditions, current medication records and laboratory findings. These conditions encompassed hypertension, coronary heart disease (CHD), diabetes, prediabetes [13, 14, 15], chronic obstructive pulmonary disease (COPD), Parkinson's disease, osteoporosis, cancer and chronic liver or kidney diseases. A comprehensive geriatric assessment (CGA) was performed using several validated instruments: Nutritional status was evaluated via the Mini Nutritional Assessment (MNA), with scores ≥ 24 indicating well‐nourished, 17–23.5 indicating malnutrition risk and < 17 indicating malnourished [16]; sleep quality was measured by the Pittsburgh Sleep Quality Index (PSQI), with a score > 10 defining poor sleep quality [17]; anxiety was assessed using the 7‐item Generalized Anxiety Disorder (GAD‐7) scale, with a score > 4 considered positive [18]; depressive symptoms were screened using the 15‐item Geriatric Depression Scale (GDS‐15), where a score ≥ 5 was indicative of depression [19]; and cognitive function was evaluated through the Montreal Cognitive Assessment (MoCA), with a score < 24 used to define mild cognitive impairment (MCI) [20].

Fasting venous blood samples were collected from all participants on the day of testing. The measured biochemical indicators included fasting blood glucose (FBG, mmol/L), glycated haemoglobin (HbA1c, %), total cholesterol (TC, mmol/L), triglycerides (TG, mmol/L), high‐density lipoprotein cholesterol (HDL‐C, mmol/L), low‐density lipoprotein cholesterol (LDL‐C, mmol/L) and interleukin‐6 (IL‐6, pg/mL). The estimated glomerular filtration rate (eGFR, mL/min/1.73 m^2^) was calculated using the Berlin Initiative Study 2 (BIS2) equation [21, 22], which is specifically designed for older adults and incorporates both serum creatinine (Scr) and serum cystatin C (CysC).

### 2.3 Quality Control

To ensure the scientific rigour and reliability of the data, the following quality control measures were implemented: (1) All research staff underwent rigorous, standardized training and evaluation. Investigators responsible for questionnaires and body composition assessments were required to demonstrate proficiency in interview techniques, scale administration and participant communication strategies. (2) High‐precision measurement instruments were utilized and calibrated according to the manufacturers' specifications prior to each use to ensure the objectivity and accuracy of the results. (3) Research data were entered into a secure database in a timely and accurate manner. Prior to statistical analysis, the dataset underwent multiple rounds of cross‐checking and verification. Randomized spot checks were performed to validate the integrity and authenticity of the recorded data.

### 2.4 Statistical Analysis

Statistical analyses were performed using SPSS version 27.0 and R software version 4.2.3. Continuous variables with a normal distribution were expressed as means ± standard deviation (X ± SD), and intergroup comparisons were conducted using independent samples *t*‐tests. Variables with a non‐normal distribution were presented as medians and interquartile ranges [M(P25, P75)], with comparisons performed using the nonparametric Kruskal–Wallis H test. Categorical variables were expressed as frequencies and percentages (*n*, %) and analysed using chi‐square (χ^2^) tests.

With 4‐year all‐cause mortality as the primary outcome, multivariable Cox proportional hazards models were employed to calculate hazard ratios (HR) and 95% confidence intervals (CI), with stepwise adjustment for baseline covariates showing significant differences. For the analysis of WHR, the values were scaled by a factor of 100 to evaluate potential non‐linear associations; RCS were utilized, complemented by piecewise linear regression models to identify specific threshold effects. Finally, survival curves were estimated using the Kaplan–Meier method, and the log‐rank test was applied to compare cumulative survival rates across different frailty statuses and body composition index levels.

## 3 Results

### 3.1 Baseline Characteristics

**FIGURE 1:**
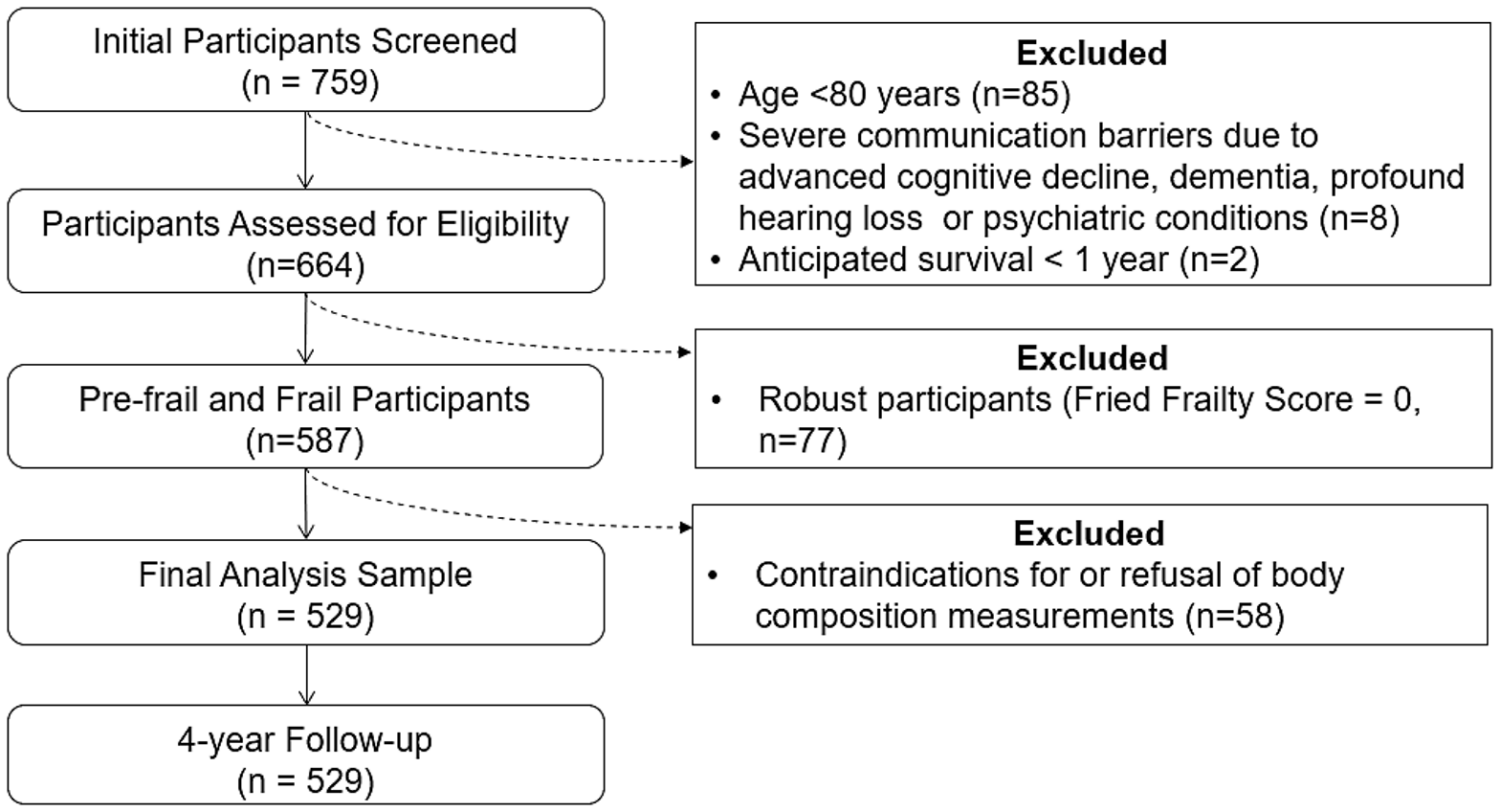
Flowchart of participant enrolment.

Among 759 initially screened participants, 95 were excluded due to age < 80 years (*n* = 85), severe communication barriers (*n* = 8) or anticipated survival < 1 year (*n* = 2). Of the 664 individuals assessed, 77 robust participants (Fried frailty score = 0) were excluded, leaving 587 prefrail and frail individuals. Further exclusion of 58 participants due to contraindications or refusal of body composition measurements yielded a final analytic sample of 529, all of whom completed the 4‐year follow‐up. The detailed flowchart is presented in Figure 1.

The median age of the cohort was 87.00 years. During a median follow‐up period of 3.84 years, 88 all‐cause mortality events (16.6%) were recorded. Participants in the death group were significantly older (median: 89 vs. 86 years, *p* < 0.001) and had a higher proportion of males (*p* < 0.001), a lower proportion of married (*p* = 0.014), a higher income status (*p* < 0.001) and a higher proportion of caregiving (*p* = 0.009). The death group exhibited higher prevalence rates of hypertension and COPD (all *p* < 0.05). Compared to survivors, those who died had higher malnutrition risk (*p* = 0.002). Notably, markers of adiposity, including BMI, PBF, VFA and WHR were significantly lower in the death group (all *p* < 0.05). SMI and lower limb muscle mass were also significantly higher in the death group (Table 1).

**TABLE 1:** Comparison of baseline characteristics between survival and death groups in the oldest‐old.

Variable	Total (*n* = 529)	Survival (*n* = 441)	Death (*n* = 88)	*t*/Z/X^2^	*p*
Age (years)	87.0 (84.0, 89.0)	86.0 (84.0, 88.0)	89.0 (86.0, 90.0)	−5.20	< 0.001
Sex (male), *n* (%)	225 (42.53)	166 (37.64)	59 (67.05)	25.95	< 0.001
Ethnicity (Han), *n* (%)	515 (97.35)	428 (97.05)	87 (98.86)	0.36	0.547
Education (college or above), *n* (%)	247 (46.69)	204 (46.26)	43 (48.86)	0.20	0.655
Marital status, *n* (%)				5.98	0.014
Married	317 (59.92)	254 (57.60)	63 (71.59)		
Divorced/widowed/single	212 (40.08)	187 (42.40)	25 (28.41)		
Monthly income, RMB				15.53	< 0.001
< 20 000	323 (61.06)	289 (65.53)	38 (43.18)		
≥ 20 000	206 (38.94)	152 (34.47)	50 (56.82)		
Caregiving status (with care), *n* (%)	428 (80.91)	348 (78.91)	80 (90.91)	6.84	0.009
Smoking, *n* (%)	8 (1.51)	7 (1.59)	1 (1.14)	0.00	1.000
Alcohol consumption, *n* (%)	158 (29.87)	124 (28.12)	34 (38.64)	3.87	0.049
Medical history, *n* (%)					
Hypertension	398 (75.24)	322 (73.02)	76 (86.36)	7.02	0.008
CHD	329 (62.19)	272 (61.68)	57 (64.77)	0.30	0.585
Diabetes mellitus status				0.22	0.898
Normal	117 (22.12)	96 (21.77)	21 (23.86)		
Prediabetes	199 (37.62)	166 (37.64)	33 (37.50)		
Diabetes	213 (40.27)	179 (40.59)	34 (38.64)		
COPD	124 (23.44)	93 (21.09)	31 (35.23)	8.17	0.004
Parkinson's disease	16 (3.02)	12 (2.72)	4 (4.55)	0.33	0.568
Chronic liver disease	67 (12.67)	52 (11.79)	15 (17.05)	1.83	0.176
Chronic kidney disease	86 (16.26)	68 (15.42)	18 (20.45)	1.37	0.242
Cancer	101 (19.09)	78 (17.69)	23 (26.14)	3.39	0.066
Osteoporosis	287 (54.25)	243 (55.10)	44 (50.00)	0.77	0.380
Polypharmacy (≥ 5 drugs), *n* (%)	357 (67.49)	290 (65.76)	67 (76.14)	3.60	0.058
History of hospitalization within the past year, *n* (%)				4.16	0.125
Never	346 (65.41)	293 (66.44)	53 (60.23)		
1–2 times	165 (31.19)	136 (30.84)	29 (32.95)		
> 2 times	18 (3.40)	12 (2.72)	6 (6.82)		
Nutritional status (MNA), *n* (%)				10.70	0.005
Malnourished	16 (3.02)	9 (2.04)	7 (7.95)		
Malnutrition risk	64 (12.10)	50 (11.34)	14 (15.91)		
Well‐nourished	449 (84.88)	382 (86.62)	67 (76.14)		
Poor sleep quality (PSQI), *n* (%)	305 (57.66)	253 (57.37)	52 (59.09)	0.09	0.765
Anxiety (GAD‐7), *n* (%)	52 (9.83)	45 (10.20)	7 (7.95)	0.42	0.518
Depression (GDS‐15), *n* (%)	28 (5.63)	26 (6.27)	2 (2.44)	1.23	0.267
Cognitive impairment (MoCA), *n* (%)	410 (77.50)	338 (76.64)	72 (81.82)	1.13	0.289
Biochemical parameters					
FBG, mmol/L	5.42 (4.97, 6.27)	5.45 (4.97, 6.27)	5.30 (5.09, 6.15)	−0.46	0.649
HbA1c, %	6.10 (5.80, 6.50)	6.10 (5.80, 6.50)	6.00 (5.80, 6.60)	−0.56	0.573
TC, mmol/L	4.28 (3.72, 5.04)	4.28 (3.70, 5.08)	4.35 (3.84, 4.73)	−0.02	0.987
TG, mmol/L	1.26 (0.93, 1.62)	1.29 (0.93, 1.67)	1.12 (0.90, 1.58)	−1.18	0.238
HDL‐C, mmol/L	1.57 (1.33, 1.94)	1.57 (1.33, 1.94)	1.61 (1.27, 1.96)	−0.01	0.994
LDL‐C, mmol/L	2.75 (2.17, 3.46)	2.75 (2.15, 3.50)	2.82 (2.39, 3.34)	−0.28	0.783
IL‐6, pg/mL	1.16 (0.64, 2.34)	1.16 (0.68, 2.25)	1.22 (0.60, 2.89)	−0.11	0.916
eGFR, mL/min/1.73m^2^	57.59 (48.71, 66.66)	58.66 (49.24, 67.17)	54.21 (44.67, 61.25)	−2.50	0.012
Physical performance					
Gait speed, m/s	0.80 ± 0.22	0.81 ± 0.21	0.71 ± 0.24	3.95	< 0.001
Handgrip strength, kg	22.50 (18.55, 26.95)	22.30 (18.60, 26.95)	22.95 (18.10, 26.77)	−0.51	0.611
FTSST, s	14.45 (11.80, 17.97)	14.42 (11.76, 17.60)	16.04 (11.80, 20.04)	−0.96	0.338
Body composition					
BMI (kg/m^2^)	23.93 ± 3.53	24.09 ± 3.54	23.16 ± 3.36	2.26	0.024
BMI classification				4.99	0.173
Underweight	29 (5.482)	21 (4.76)	8 (9.09)		
Normal	238 (44.99)	198 (44.90)	40 (45.46)		
Overweight	198 (37.43)	164 (37.19)	34 (38.64)		
Obesity	64 (12.10)	58 (13.15)	6 (6.82)		
PBF (%)	32.80 (27.40, 37.60)	33.10 (28.20, 37.90)	29.95 (23.38, 34.33)	−4.07	< 0.001
VFA (cm^2^)	105.30 (78.90, 136.40)	106.80 (81.40, 140.80)	98.65 (69.20, 125.60)	−2.83	0.005
WHR (%)^a^	89.00 (83.00, 94.00)	89.00 (84.00, 94.00)	86.50 (81.00, 92.00)	−3.03	0.002
SMI (kg/m^2^)	6.40 (5.70, 7.10)	6.30 (5.70, 7.00)	6.50 (5.97, 7.20)	−2.28	0.023
Muscle mass (kg)					
Trunk	17.90 (16.00, 20.30)	17.80 (15.90, 20.10)	18.60 (16.88, 20.92)	−1.78	0.075
Right upper limb	2.02 (1.71, 2.42)	1.99 (1.71, 2.40)	2.13 (1.85, 2.51)	−1.58	0.114
Left upper limb	1.96 (1.68, 2.35)	1.94 (1.65, 2.35)	2.06 (1.79, 2.46)	−1.72	0.086
Right lower limb	6.11 (5.19, 7.32)	5.99 (5.14, 7.11)	6.92 (5.57, 7.89)	−3.52	< 0.001
Left lower limb	6.05 (5.23, 7.27)	5.95 (5.16, 7.05)	6.92 (5.63, 7.94)	−3.88	< 0.001

Abbreviations: BMI, body mass index; CHD, coronary heart disease; COPD, chronic obstructive pulmonary disease; eGFR, estimated glomerular filtration rate; FBG, fasting blood glucose; GAD‐7, 7‐item Generalized Anxiety Disorder scale; GDS‐15, 15‐item Geriatric Depression Scale; HbA1c, glycated haemoglobin; HDL‐C, high‐density lipoprotein cholesterol; IL‐6, interleukin‐6; LDL‐C, low‐density lipoprotein cholesterol; MoCA, Montreal Cognitive Assessment; PBF, percent body fat; PSQI, Pittsburgh Sleep Quality Index; SMI, skeletal muscle index; TC, total cholesterol; TG, triglycerides; VFA, visceral fat area; WHR, waist‐to‐hip ratio.

^a^ WHR was entered into the model as a percentage (original value × 100).

### 3.2 Cox Proportional Hazards Regression Analysis of Adipose Reserves and Long‐Term Mortality Risk

To evaluate whether frailty status moderates the association between adipose reserves and mortality, stratified multivariate Cox regression analyses were performed (Table 2).

**TABLE 2:** Multivariate cox proportional hazards regression analysis of body composition and mortality in older adults living with prefrailty.

Variable	Model 1		Model 2		Model 3	
	HR (95% CI)	*p*	HR (95% CI)	*p*	HR (95% CI)	*p*
BMI (kg/m^2^)	0.90 (0.82–0.99)	0.023	0.90 (0.82–0.99)	0.023	0.94 (0.83–1.05)	0.266
PBF (%)	0.92 (0.88–0.96)	< 0.001	0.91 (0.88–0.96)	< 0.001	0.92 (0.87–0.98)	0.006
VFA (cm^2^)	0.98 (0.97–0.99)	< 0.001	0.98 (0.97–0.99)	< 0.001	0.99 (0.97–0.99)	0.013
WHR (%)^a^	0.91 (0.87–0.94)	< 0.001	0.90 (0.86–0.94)	< 0.001	0.91 (0.86–0.95)	< 0.001
SMI (kg/m^2^)	0.91 (0.65–1.28)	0.604	0.92 (0.66–1.28)	0.618	0.92 (0.60–1.41)	0.712
Muscle mass (kg)						
Trunk	0.90 (0.79–1.01)	0.072	0.89 (0.79–1.01)	0.065	0.87 (0.76–1.01)	0.069
Right upper limb	0.50 (0.24–1.04)	0.064	0.49 (0.24–1.03)	0.061	0.49 (0.20–1.19)	0.116
Left upper limb	0.50 (0.24–1.06) 0.50 (0.24–1.06)	0.071	0.49 (0.23–1.06)	0.069	0.50 (0.21–1.21)	0.125
Right lower limb	1.02 (0.78–1.34)	0.859	1.02 (0.78–1.35)	0.870	0.84 (0.60–1.19)	0.328
Left lower limb	1.01 (0.77–1.33)	0.950	1.01 (0.76–1.33)	0.947	0.85 (0.60–1.20)	0.358

*Note:* Model 1: Adjusted for age and sex. Model 2: Adjusted for Model 1 + marital status, caregiving status, income and alcohol consumption history. Model 3: Adjusted for Model 2 + hypertension, COPD, nutritional status, gait speed and eGFR.

Abbreviations: BMI, body mass index; CI, confidence interval; FTSST, Five‐Times Sit‐to‐Stand Test; HR, hazard ratio; PBF, percent body fat; SMI, skeletal muscle index; VFA, visceral fat area; WHR, waist‐to‐hip ratio.

^a^ WHR was entered into the model as a percentage (original value × 100).

In the fully adjusted model (Model 3, controlling for age, sex, comorbidities and nutritional status), higher adipose indicators demonstrated a significant protective effect. Every 1% increase in PBF was associated with a 6% reduction in mortality risk (HR = 0.92, 95% CI: 0.87–0.98, *p* = 0.006). Similarly, VFA (HR = 0.99, 95% CI: 0.97–0.99, *p* = 0.013) and WHR (HR = 0.91, 95% CI: 0.86–0.95, *p* < 0.001) were inversely associated with all‐cause mortality. No significant independent associations were found for SMI or muscle mass.

However, the survival‐protective effects of adipose were not significant in the frail subgroup. In Model 3, BMI, PBF, VFA and WHR showed no independent correlation with mortality (all *p* > 0.05, Table 3).

**TABLE 3:** Multivariate Cox proportional hazards regression analysis of body composition and mortality in older adults living with frailty.

Variable	Model 1		Model 2		Model 3	
	HR (95% CI)	*p*	HR (95% CI)	*p*	HR (95% CI)	*p*
BMI (kg/m^2^)	0.97 (0.89–1.06)	0.466	0.98 (0.90–1.07)	0.694	0.90 (0.72–1.12)	0.334
PBF (%)	0.99 (0.95–1.03)	0.499	0.99 (0.95–1.04)	0.759	0.91 (0.82–1.01)	0.074
VFA (cm^2^)	1.00 (0.99–1.01)	0.995	1.00 (0.99–1.01)	0.778	0.99 (0.98–1.01)	0.422
WHR (%)^a^	0.99 (0.95–1.03)	0.694	1.00 (0.95–1.04)	0.840	0.96 (0.89–1.04)	0.333
SMI (kg/m^2^)	1.00 (0.70–1.44)	0.989	1.05 (0.73–1.52)	0.783	1.48 (0.64–3.42)	0.356
Muscle mass (kg)						
Trunk	0.99 (0.88–1.11)	0.898	1.01 (0.90–1.13)	0.898	1.05 (0.82–1.36)	0.694
Right upper limb	0.96 (0.48–1.93)	0.915	1.04 (0.52–2.08)	0.901	1.39 (0.29–6.72)	0.680
Left upper limb	1.13 (0.60–2.14)	0.701	1.20 (0.63–2.29)	0.575	1.32 (0.29–5.94)	0.720
Right lower limb	0.99 (0.77–1.28)	0.965	1.04 (0.80–1.35)	0.793	1.10 (0.59–2.03)	0.772
Left lower limb	1.09 (0.84–1.42)	0.513	1.14 (0.87–1.49)	0.345	1.75 (0.93–3.26)	0.080

*Note:* Model 1: Adjusted for age and sex. Model 2: Adjusted for Model 1 + marital status, caregiving status, income and alcohol consumption history. Model 3: Adjusted for Model 2 + hypertension, COPD, nutritional status, gait speed and eGFR.

Abbreviations: BMI, body mass index; CI, confidence interval; HR, hazard ratio; PBF, percent body fat; SMI, skeletal muscle index; VFA, visceral fat area; WHR, waist‐to‐hip ratio.

^a^ WHR was entered into the model as a percentage (original value × 100).

### 3.3 Non‐Linear and Threshold Effect Analysis of Adipose Indicators

To further investigate the precise quantitative relationship between adipose reserve indicators (PBF, VFA and WHR) and the risk of all‐cause mortality, we employed RCS and piecewise regression models for analysis.

**FIGURE 2:**
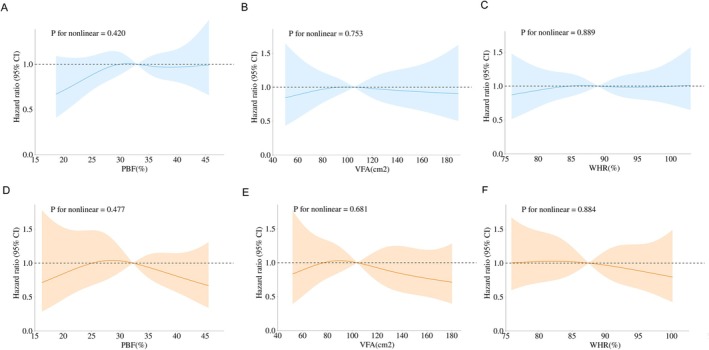
RCS curves for the associations between adipose reserve indicators and all‐cause mortality risk.

RCS analysis, adjusted for multiple covariates, showed that the non‐linear test results for PBF, VFA and WHR were not significant in either the prefrailty (*p* non‐linear > 0.05, Figure 2A–C) or the frailty subgroup (*p* non‐linear > 0.05, Figure 2D–F).

Threshold analysis conducted in the prefrailty group (Table S1) further confirmed that there were no significant threshold effects between adiposity reserve indicators and mortality risk (likelihood ratio test *p* > 0.05). Although potential inflection points were initially detected for PBF at 24.60%, VFA at 159.02 cm^2^ and WHR at 0.99, the two‐piecewise linear regression model offered no statistical advantage over the standard linear regression model. Notably, no deaths were observed among the few individuals with a VFA ≥ 159.02 cm^2^, and only two deaths occurred in the stratum with a WHR ≥ 0.99; some threshold estimates exhibited wide confidence intervals.

In the frailty group, threshold analysis further verified that there was no significant association between adipose reserve indicators (PBF, VFA and WHR) and mortality risk, nor were there any significant threshold effects (likelihood ratio test *p* > 0.05).

Interaction analyses revealed that frailty status significantly modified the associations between adiposity reserve indicators and all‐cause mortality risk (Table 4). Significant interactions with frailty status were observed for PBF (*p* for interaction = 0.018), VFA (*p* for interaction = 0.028) and WHR (*p* for interaction = 0.019).

**TABLE 4:** Interaction analyses of the associations between adiposity indicators and all‐cause mortality risk by frailty status.

Outcome	HR	*p*	*p* for interaction
PBF			0.018
Prefrail	0.92 (0.87–0.98)	0.006	
Frail	0.91 (0.82–1.01)	0.074	
VFA			0.028
Prefrail	0.99 (0.97–1.00)	0.013	
Frail	0.99 (0.98–1.01)	0.422	
WHR			0.019
Prefrail	0.91 (0.86–0.95)	< 0.001	
Frail	0.96 (0.89–1.04)	0.333	

*Note:* Adjusted for age, sex, marital status, caregiving status, income, alcohol consumption history, hypertension, COPD, nutritional status, gait speed and eGFR.

In the prefrailty subgroup, higher levels of adiposity indicators were consistently and significantly associated with a decreased risk of all‐cause mortality. Specifically, each unit increase in PBF, VFA and WHR was associated with an 8% (HR = 0.92, 95% CI: 0.87–0.98, *p* = 0.006), 1% (HR = 0.99, 95% CI: 0.97–1.00, *p* = 0.013) and 9% (HR = 0.91, 95% CI: 0.86–0.95, *p* < 0.001) reduction in mortality risk, respectively. Conversely, within the frailty subgroup, these protective associations were entirely attenuated and statistical significance was lost for all three indicators (PBF: HR = 0.91, 95% CI: 0.82–1.01, *p* = 0.074; VFA: HR = 0.99, 95% CI: 0.98–1.01, *p* = 0.422; WHR: HR = 0.96, 95% CI: 0.89–1.04, *p* = 0.333).

### 3.4 Kaplan–Meier Survival Analysis of Adipose Reserves and All‐Cause Mortality

**FIGURE 3:**
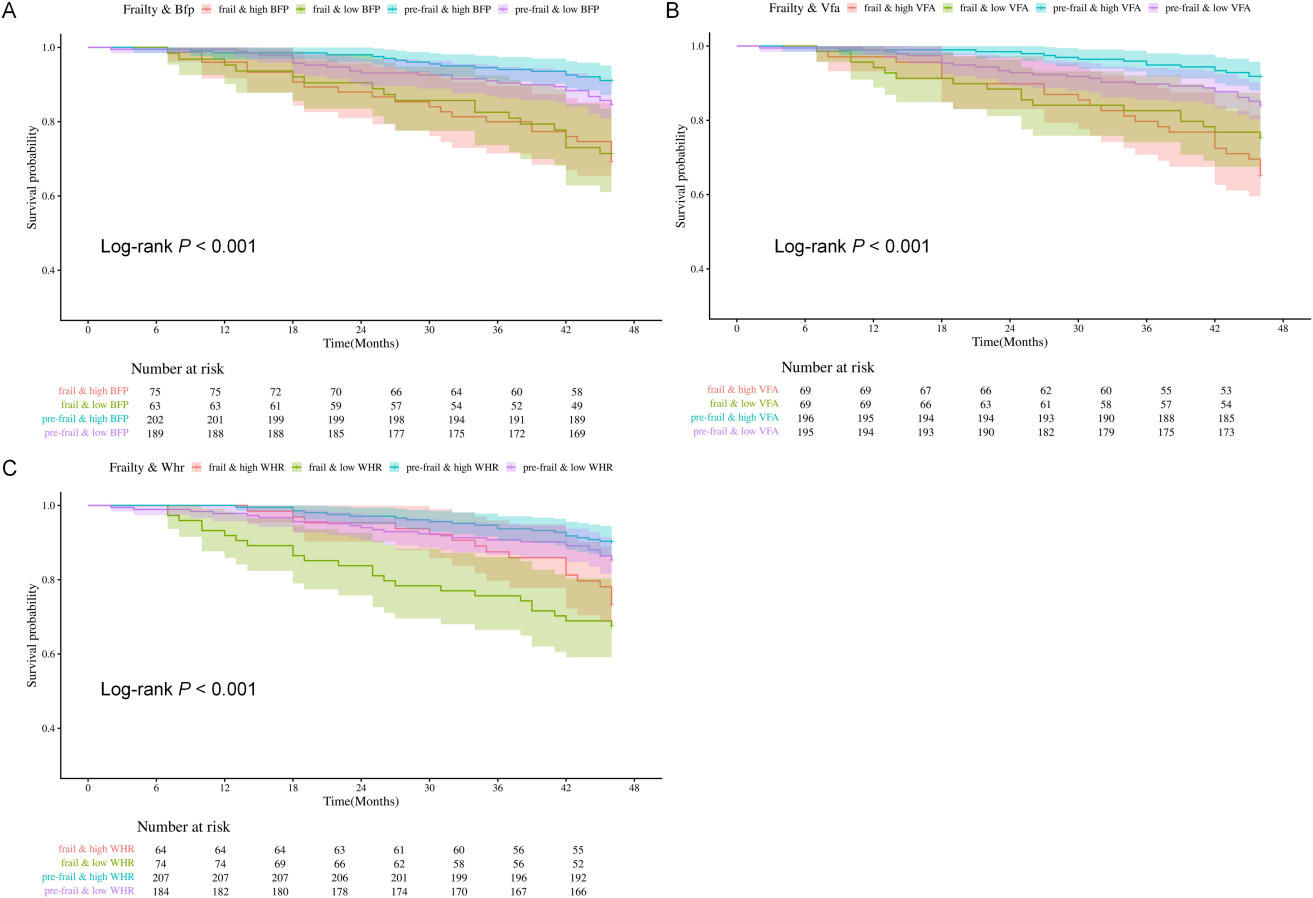
Kaplan–Meier survival curves for all‐cause mortality stratified by frailty status and adipose reserve indicators. (A) Body fat percentage (BFP); (B) visceral fat area (VFA); (C) waist‐to‐hip ratio (WHR). Participants were categorized into four groups based on the combination of frailty status (frail vs. prefrail) and body composition levels (high vs. low, defined by the median values). Survival probabilities were compared using the log‐rank test (all *p* < 0.001). The numbers at risk for each group at different time points are shown below the x‐axis.

Given the absence of non‐linear correlations or significant threshold effects between adipose reserve indicators and mortality risk, participants were stratified into high and low groups based on the median values of each metric. Kaplan–Meier survival curves and Log‐rank tests revealed that the impact of adipose reserves on survival followed distinct trajectories depending on frailty status, exhibiting a significant interaction with functional reserves (*p* < 0.001, Figure 3 and Table 5).

**TABLE 5:** Comparison of 4‐year survival rates across different groups stratified by frailty status and adipose levels.

Variables	*N*	Events	Rate/1000 (person‐years)	Log‐rank *p*
Frailty and PBF				< 0.001
Frail and high PBF	75	23	79.86	
Frail and low PBF	63	18	74.40	
Prefrail and high PBF	202	18	23.21	
Prefrail and low PBF	189	29	39.96	
Frailty and VFA				< 0.001
Frail and high VFA	69	24	90.58	
Frail and low VFA	69	17	64.16	
Prefrail and high VFA	196	16	21.26	
Prefrail and low VFA	195	31	41.40	
Frailty and WHR				< 0.001
Frail and high WHR	64	17	69.17	
Frail and low WHR	74	24	84.46	
Prefrail and high WHR	207	20	25.16	
Prefrail and low WHR	184	27	38.21	

## 4 Discussion

Based on a 4‐year prospective follow‐up, this study identified adipose reserve indicators, including PBF, VFA and WHR, as factors inversely associated with long‐term all‐cause mortality in the oldest‐old. Importantly, our study reveals that this association is significantly modulated by frailty status: The survival benefits associated with higher adipose metrics are prominent primarily in the prefrail stage, whereas this protective effect diminishes when clinical frailty is established.

A previous prospective study involving Mexican Americans aged 75 years and older reported that a higher BMI was significantly associated with a reduced risk of 12‐year all‐cause mortality [23]. However, BMI remains a non‐specific metric that fails to distinguish between adipose tissue and skeletal muscle, thus potentially masking the distinct biological contributions of different body components to survival. By utilizing body composition analysis, our study further extends these findings. In advanced ageing, the geriatric organism is generally in a state of chronic hypermetabolism and covert consumption. In this study, a significant inverse correlation was observed between adipose metrics (PBF, VFA and WHR) and mortality risk among the prefrail population. Based on these findings, we speculate that during this specific stage, moderately abundant adipose reserves not only reflect a superior level of anabolic metabolism and robust energy turnover potential but are also highly likely to serve as an active, adaptive defence mechanism established by the geriatric organism during age‐related remodelling [24, 25]. As a plausible biological hypothesis, when facing acute clinical stressors such as pulmonary infections or fractures, these energy stores might effectively mitigate the catabolic breakdown of skeletal muscle proteins, thereby acting as a critical metabolic buffer that may support survival during acute clinical stressors.

Understandably, the intrinsic relationship between adiposity and survival in advanced ageing is multifaceted and complex [26, 27, 28]. While acknowledging the potential biological protective effects of adipose tissue, we should also objectively consider the dual mechanisms underlying this inverse correlation from an epidemiological perspective. On one hand, higher WHR, VFA and PBF may partly serve as favourable phenotypic indicators of better overall nutritional status, adequate caloric intake and the absence of entering pathological wasting states such as occult diseases or pre‐cachexia, thereby minimizing the confounding effects of reverse causality in clinical observations. On the other hand, the energy reserve effect inherent to these adipose metrics indeed provides substantial metabolic support to the organism. Therefore, the survival advantage associated with higher adiposity in the prefrail oldest‐old is more likely a comprehensive manifestation of both a favourable baseline nutritional status and the active metabolic buffering capacity provided by adequate fat mass.

Frailty status significantly modulates the association between adipose reserves and survival outcomes. A large‐scale cohort study of 29 937 older adults demonstrated that being overweight or having Grade 1 obesity serves as a protective factor against mortality in individuals with mild‐to‐severe frailty, whereas Grades 2 and 3 obesity are associated with increased mortality risk in nonfrail populations [29]. Our findings in the oldest‐old align with this evidence, further confirming the robust protective effect of adipose reserves during the prefrailty stage. However, this protective effect does not persist indefinitely. When the organism is excessively depleted and enters a state of decompensated frailty, the metabolic buffering capacity of fat diminishes, and its protective effect vanishes. From a biological standpoint, we hypothesize that this phenomenon may be related to the emergence of metabolic dysfunction and inflammaging as individuals transition further along the frailty spectrum [30]. Under this plausible theoretical framework, severe mitochondrial dysfunction might hinder the effective conversion of triglycerides stored in adipose tissue into bioavailable energy, potentially resulting in a metabolic stalemate where the organism possesses fat reserves but lacks the capacity to efficiently utilize them. More critically, it is conceivable that the adipose tissue undergoes a phenotypic shift, transforming from a primary energy reservoir into a source of pro‐inflammatory cytokines, such as IL‐6 and TNF‐α [31, 32]. This persistent systemic inflammation could further inhibit protein synthesis and exacerbate organ damage, ultimately offering a potential explanation for why the survival benefits of fat as an energy reserve are offset during advanced frailty [33].

Notably, our analysis revealed no independent predictive role for SMI or skeletal muscle mass regarding mortality risk in this cohort. While seemingly a nonsignificant result, this lack of association further underscores the potentially paramount importance of adipose reserves for survival in the oldest‐old, relative to muscle mass alone [34]. In the presence of acute stressors, the metabolic buffering capacity of adipose tissue may be a critical, albeit nonexclusive, factor influencing short‐term survival trajectories [35, 36]. We speculate that under conditions of severely limited physiological reserves, the geriatric organism may prioritize the maintenance of adipose‐driven metabolic homeostasis when physiological reserves are severely limited, potentially improving short‐term survival prospects.

Beyond skeletal muscle mass, we incorporated functional indicators into our analysis, specifically examining handgrip strength for upper limb power and the FTSST for lower limb function. Gait speed, as a more sensitive performance metric, exhibited a significant intergroup difference and was subsequently adjusted for in our Cox proportional hazards models. Nevertheless, baseline physical function failed to demonstrate a robust independent correlation with long‐term mortality risk. This lack of strong correlation may be largely attributed to the high heterogeneity of retrospective or ongoing lifestyle modalities in this extreme longevity cohort. In the absence of structured, supervised resistance training, diet‐induced weight loss or severe nutritional restrictions in the oldest‐old invariably lead to a concurrent depletion of skeletal muscle, fat mass and bone mineral density [37, 38]. This structural attrition inevitably exacerbates age‐related sarcopenia and compounding frailty outcomes, thereby counteracting the potential survival benefits initially offered by superior functional capacity. Consequently, the limited prognostic predictive value of physical function captured in this study does not diminish its clinical relevance; rather, it highlights that in the advanced stages of ageing, individual survival trajectories may be more closely tied to energy‐reserve resilience than to motor performance alone in this population.

Although this study reveals the significant protective role and stage‐specific characteristics of adipose reserves for long‐term survival in the oldest‐old, several limitations must be acknowledged. First, the sample size of this study is relatively limited, and all participants were recruited from a community setting within a single region. Although the current sample size is sufficient to support stratified analyses and interaction tests, larger, multicentre cohorts of the oldest‐old, encompassing a broader range of geographic and ethnic backgrounds, are required in the future to further validate these findings. Second, the use of BIA has inherent limitations, as age‐related changes in body water distribution, hydration abnormalities, subclinical oedema and altered tissue conductivity may compromise its accuracy. Furthermore, relying solely on baseline assessments of body composition and frailty—without tracking their longitudinal trajectories—restricts our ability to draw robust causal inferences regarding dynamic body composition changes and long‐term survival. Third, frailty is intricately linked to complex metabolic disturbances, which are closely intertwined with the homeostasis of both adipose and skeletal muscle tissues. Although we adjusted for key covariates, we lacked detailed metabolic assessments. Future studies incorporating these metrics are needed to elucidate the precise metabolic mechanisms underlying frailty. Furthermore, the landscape of geriatric pharmacotherapy is rapidly evolving. In recent years, novel medications such as glucagon‐like peptide‐1 receptor agonists have been increasingly prescribed for diabetes and weight management. Since their use was negligible in our 2019 cohort, we could not address this issue; however, we suggest that future cohort studies of the oldest‐old systematically collect data on these medications to clarify their long‐term effects on frailty and survival.

In conclusion, adipose reserve indicators are inversely associated with all‐cause mortality among prefrail oldest‐old individuals, highlighting the biological value of metabolic reserves in buffering acute clinical stressors. However, this protective effect is absent in the frail population. Consequently, clinical weight management for the oldest‐old should move away from indiscriminate fat reduction. Instead, clinicians must meticulously evaluate individual frailty status, prioritize the preservation of muscle mass and function, and guarantee adequate nutritional reserves to optimize survival outcomes.

## Funding

The study was supported by Key Research and Development Program of Shandong Province (2024CXGC010411), Healthcare Fund (24BJZ26) and Key Research and Development Plan of Ningxia Hui Autonomous Region (2024BEG02032).

## Ethics Statement

This study received approval from the Research Ethics Committee of the Chinese PLA General Hospital (Ethics Number: S2018‐102‐02) and was registered with the China Clinical Trial Registry (https://www.chictr.org.cn) on 17 April 2019 (Registration No. ChiCTR900022576). The study objectives were thoroughly explained to all participants, and their informed written consent was obtained. We also ensured that this study strictly adhered to the guidelines and regulations of the Declaration of Helsinki.

## Consent

Written informed consent was obtained from all participants.

## Conflicts of Interest

The authors declare no conflicts of interest.

## Supporting information


**Table S1:** Two‐piecewise linear regression analysis of the threshold effects of adipose reserve indicators on all‐cause mortality risk among the oldest‐old.

## Data Availability

The datasets used and analysed in this study are available from the corresponding author on reasonable request.
