# Supplementary material for: Adipose Reserves and Survival: Protective in Prefrail but Ineffective in Frail Oldest‐Old
Source: J Cachexia Sarcopenia Muscle. 2026 Aug 13;17(4):e70358. doi: 10.1002/jcsm.70358 (PMC13473701; doi:10.1002/jcsm.70358)
Supplement: Supplementary file 1 [file JCSM-17-e70358-s001.docx]

**Table S1 Two-piecewise linear regression analysis of the threshold effects of adipose reserve indicators on all-cause mortality risk among the oldest-old.**

| **Pre-frail** | | | **Frail** | | |
| --- | --- | --- | --- | --- | --- |
| Outcome | effect | *P* | Outcome | effect | *P* |
| **PBF** |  |  | **PBF** |  |  |
| Inflection point | 24.60 |  | Inflection point | 23.29 |  |
| <24.60 | 0.70 (0.46 - 1.05) | 0.083 | <23.29 | 0.75 (0.49 - 1.15) | 0.185 |
| ≥24.60 | 0.96 (0.88 - 1.04) | 0.323 | ≥23.29 | 1.03 (0.96 - 1.10) | 0.468 |
| P for likelihood test |  | 0.174 | P for likelihood test |  | 0.146 |
| **VFA** |  |  | **VFA** |  |  |
| Inflection point | 159.02 |  | Inflection point | 138.90 |  |
| <159.02 | 0.99 (0.98 - 1.00) | 0.081 | <138.90 | 1.01 (1.00 - 1.03) | 0.084 |
| ≥159.02 | No events |  | ≥138.90 | 0.92 (0.82 - 1.03) | 0.127 |
| P for likelihood test |  | 0.280 | P for likelihood test |  | 0.101 |
| **WHR** |  |  | **WHR** |  |  |
| Inflection point | 99.00 |  | Inflection point | 73.50 |  |
| <99.00 | 0.90 (0.85 - 0.95) | <0.001 | <73.50 | 1.00 (0.00 - Inf) | 1.000 |
| ≥99.00 | 0.09 (0.00 - Inf) | 1.000 | ≥73.50 | 0.92 (0.82 - 1.02) | 0.126 |
| P for likelihood test |  | 0.752 | P for likelihood test |  | 0.848 |

**Notes:** Adjusted for age, sex, marital status, caregiving status, income, alcohol consumption history, hypertension, COPD, ,nutritional status, gait speed and eGFR.
